# Effect of a combination of genistein, polyunsaturated fatty acids and vitamins D3 and K1 on bone mineral density in postmenopausal women: a randomized, placebo-controlled, double-blind pilot study

**DOI:** 10.1007/s00394-012-0304-x

**Published:** 2012-02-03

**Authors:** Joan Lappe, Iris Kunz, Igor Bendik, Kevin Prudence, Peter Weber, Robert Recker, Robert P. Heaney

**Affiliations:** 1 Osteoporosis Research Center, Creighton University Medical Center, 601 North 30th Street, Suite 4820, Omaha, NE 68131 USA; 2 DSM Nutritional Products, R&D Human Nutrition and Health, Kaiseraugst, Switzerland

**Keywords:** Genistein, Osteoporosis, Clinical trial, Isoflavones, Bone mineral density, Safety

## Abstract

**Purpose:**

Many postmenopausal women desire non-pharmaceutical alternatives to hormone therapy for protection against osteoporosis. Soybean isoflavones, especially genistein, are being studied for this purpose. This study examined the effects of synthetic genistein in combination with other potential bone-protective dietary molecules on bone mineral density (BMD) in early postmenopausal women.

**Methods:**

In this 6-month double-blind pilot study, 70 subjects were randomized to receive daily either calcium only or the geniVida™ bone blend (GBB), which consisted of genistein (30 mg/days), vitamin D3 (800 IU/days), vitamin K1 (150 μg/days) and polyunsaturated fatty acids (1 g polyunsaturated fatty acids as ethyl ester: eicosapentaenoic acid/docosahexaenoic acid ratio = ~2/1). Markers of bone resorption and formation and BMD at the femoral neck, lumbar spine, Ward’s triangle, trochanter and intertrochanter, total hip and whole body were assessed.

**Results:**

Subjects supplemented with the GBB (*n* = 30) maintained femoral neck BMD, whereas in the placebo group (*n* = 28), BMD significantly decreased (*p* = 0.007). There was also a significant difference (*p* < 0.05) in BMD between the groups at Ward’s triangle in favor of the GBB group. Bone-specific alkaline phosphatase and N-telopeptide significantly increased in the GBB group in comparison with those in baseline and in the placebo group. The GBB was well tolerated, and there were no significant differences in adverse events between groups.

**Conclusions:**

The GBB may help to prevent osteoporosis and reduce fracture risk, at least at the hip, in postmenopausal women. Larger and longer-term clinical trials are warranted.

## Introduction

In response to declining estrogen levels, women can lose substantial amounts of bone mass in the decade following menopause, which markedly increases their fracture risk [1]. Until 2002, postmenopausal women were typically prescribed hormone therapy (HT) if they were considered to be at risk of developing osteoporosis. Although research from the Women’s Health Initiative Trial confirmed that HT reduces postmenopausal bone loss and hip fracture risk [2], the results, along with findings from several other large-scale studies, have also raised safety concerns about the use of HT [3, 4]. These concerns have led to a dramatic decline in HT use [5] and the need to identify non-hormonal anti-osteoporotic agents.

One widely studied non-pharmaceutical alternative for promoting postmenopausal bone health is isoflavones; among commonly consumed foods, they are found in physiologically relevant amounts only in the soybean and soyfoods. Isoflavones are present in soybeans almost exclusively as glycosides, and the three aglycone isoflavones genistein, daidzein and glycitein and their respective glycosides account for approximately 50, 40 and 10% of total isoflavone content, respectively [6]. Mean isoflavone intake in western countries is typically <3 mg/days [7], whereas in Japan, daily intake of approximately 30–50 mg can be achieved [8].

In general, isoflavones are considered to be phytoestrogens, because some members were shown to bind to transactivate estrogen receptors and to initiate gene expression [9]. Initial speculation about their efficacy was based on their estrogen-like properties and early research showing that the chemically synthesized isoflavone structurally derivatized drug, ipriflavone, exerted skeletal benefits [10]. The results of prospective epidemiologic studies conducted in Shanghai [11] and Singapore [12] seem to support the efficacy of isoflavones, as high soy intake in these studies was associated with approximately 30% reductions in fracture risk. However, the >25 clinical trials that have examined the effects of isoflavones on bone mineral density (BMD) have produced mixed results, although two out of three recently published meta-analyses found that soy isoflavones reduced bone loss at the lumbar spine [13–15]. However, only four studies, the 3-year Italian trial by Marini et al. [16], the 2- and 3-year US studies by Levis et al. [17] and Alekel et al. [18], respectively, and the 2-year Taiwanese study by Tai et al. [19] were more than 1 year in duration. In the study by Marini et al. [16], there were dramatic increases in postmenopausal spinal and hip BMD after genistein supplementation, whereas in the other three studies, there was little evidence that soy isoflavones produced skeletal benefits [17–19].

To substantially reduce the risk of a chronic disease such as osteoporosis, through lifestyle and dietary intervention, requires the adoption of a comprehensive approach. Evidence suggests that a combination of potentially bone-protective dietary agents working through different mechanisms is more likely to result in a substantial benefit than any single agent alone. For example, dietary protein is viewed as beneficial for bone when sufficient dietary calcium is consumed, but possibly harmful when it is not [20]. Also, vitamin D enhances the absorption of calcium and may have independent skeletal benefits [21]. There is also evidence that vitamin K is needed for γ-carboxylation of specific glutamic acids which converts 3 glutamic acid (Glu) residues in osteocalcin (OC) to γ-carboxyglutamic acid (Gla) [22, 23], an essential structural modification for the integration of osteocalcin into the bone matrix. Finally, supplementation with long-chain omega-3 fatty acids eicosapentaenoic acid (EPA) and docosahexaenoic acid (DHA) may reduce bone loss [24]. In mice, isoflavones and fish oil additively induced parameters of bone structure and increased bone mass synergistically in an ovariectomy-induced bone loss model [22].

It is reasonable to speculate that when a combination of agents is used, the amount of isoflavones required for efficacy may be reduced. Therefore, we decided to conduct a pilot study using geniVida™ to determine whether a physiological dose of genistein (30 mg/days), when combined with other bioactives, will favorably impact bone health in postmenopausal women. This dose of genistein is in line with the mean intake of genistein among older Japanese following a traditional diet [8]. This pilot intervention study was conducted to generate data on the impact of a genistein bone blend (GBB) on bone loss in early postmenopausal women. Should efficacy be demonstrated, the data could be used as a basis for designing a larger and longer follow-up study.

## Subjects and methods

### Study design and trial supplementation

In this randomized, double-blind, placebo-controlled, 6-month pilot intervention study, the effect of a combination of genistein (30 mg/days), vitamin D3 (800 IU/days), vitamin K1 (150 μg/days) and polyunsaturated fatty acids 1 g (PUFAs) as ethyl ester: EPA/DHA ratio = ~2/1) on bone health in postmenopausal women was investigated. The protocol, informed consent form and advertisement for subjects were approved by the Creighton University institutional Review Board (IRB) (no. 06–14202). The study was conducted in accordance with Good Clinical Practice Guidelines from the International Conference of Harmonization and was registered at ClinicalTrial.gov (NCT 00698984).

Genistein, vitamin K1 and D3 and PUFAs were manufactured by DSM Nutritional Products, Ltd. (Kaiseraugst, Switzerland), and capsule production (active and placebo) as well as packaging and labeling took place at Intergel Division, IVC Industries Inc., NJ (USA), under GMP requirements and control. One soft-gelatin geniVida™ bone blend (GBB) capsule contained 15 mg genistein (geniVida 99.1% genistein), 500 mg PUFAs (ROPUFA^®^ 75 n-3 Ethyl Ester), 75 μg vitamin K1 (99.7% phylloquinone) and 400 IU vitamin D3 (100% cholecalciferol) together with corn oil and bees wax. Placebo capsules contained only corn oil and bees wax.

Two capsules were taken per day in the morning together with breakfast. In addition, one calcium carbonate tablet containing 500 mg elemental calcium (Ost-Cal 500, Goldine) was taken daily with either the placebo or GBB. Supplementation began on the day after the baseline visit (visit 1) and ended the morning before visit 3, 6 months after visit 1.

#### Randomization and blinding

Four-block randomization was performed by an employee of DSM Nutritional Products Ltd., who was not involved in the study. The randomization list was provided to Intergel Division, IVC Industries Inc., for packaging and placement of capsules into bottles and bottle labeling. Bottles with randomization code numbers, which were shipped to Creighton University, were dispensed to study subjects in sequenced numbers. Unblinding occurred after all data management procedures were completed. Only emergency envelopes were located at the study site. The randomization code was kept locked at the safety management company (United BioSource Corporation (UBC), Geneva, Switzerland). Subject identification was written on the appropriate bottles and entered into the subject enrollment log along with the randomization code.

#### Compliance

Study personnel dispensed supplements together with a personal diary in which the subjects documented the date and time of supplement use. Compliance was assessed on the basis of pill count. Subjects were considered compliant if 80% of the required number of pills was taken. Plasma genistein concentrations measured at baseline and at 3 and 6 months were used as a secondary confirmation of compliance but were not used as a basis for classifying subjects as compliant or non-compliant.

#### Procedures

Before the subjects were invited for the pre-study examination, a brief telephone screen was conducted to determine whether they were within the study age range, ≥1 year postmenopausal, and satisfied other inclusion/exclusion criteria. Candidates who successfully completed the telephone screening interview were scheduled for the pre-study examination. In the pre-study examination, which took place 1–2 months prior to study start, subject eligibility was again determined. Subjects signed an informed consent form prior to the pre-study examination being performed.

After successful completion of the pre-study examination, volunteers were enrolled into the study, starting with a 2-week run-in period during which time they received the placebo. During the run-in period, the volunteers familiarized themselves with the study procedures and the dietary guidelines to which they were expected to adhere. If the subjects successfully completed the run-in, they were asked to sign a second informed consent for study participation as well as to allow disclosure of personal data. They were then randomly enrolled into either the placebo or GBB group and provided the appropriate capsules.

Each subject had 5 visits (pre-study examination, start of run-in, visits 1, 2 and 3) and received 6 phone calls (screening and months 1, 2, 4 5 and follow-up) over a period of 7–9 months. They were instructed to maintain their normal diet and exercise routine. Telephone calls between visits allowed compliance to study protocol to be assessed. Fifteen days after the final visit, subjects were contacted by phone to identify any changes in health status since the final visit.

At visits 1, 2 and 3, bone markers (see below), plasma genistein, 25-hydroxyvitamin D3 (25(OH)D) and phylloquinone concentrations, dietary records and physical activity levels were assessed, and diet counseling was conducted. BMD was measured at baseline and visit 3 and safety parameters at screening and visit 3.

### Study subjects, recruitment, selection and disposition

Subjects were recruited between January 2007 and April 2008 by the research team from the Creighton University Osteoporosis Research Center. Eligibility was based on inclusion/exclusion criteria determined by physical examination, medical history, electrocardiogram (ECG), mammogram, trans-vaginal ultrasound, BMD, clinical laboratory, serology and drug and thrombophilia screening. Inclusion criteria were healthy early postmenopausal women between the ages of 45 and 55 years, 1–3 years since the last spontaneous menstrual bleeding and follicle-stimulating hormone (FSH) and 17β-estradiol (E2) concentrations >75 IU/mL and <20 ng/L, respectively, natural menopause or total hysterectomy, and smoking <10 cigarettes/days.

**Fig. 1:**
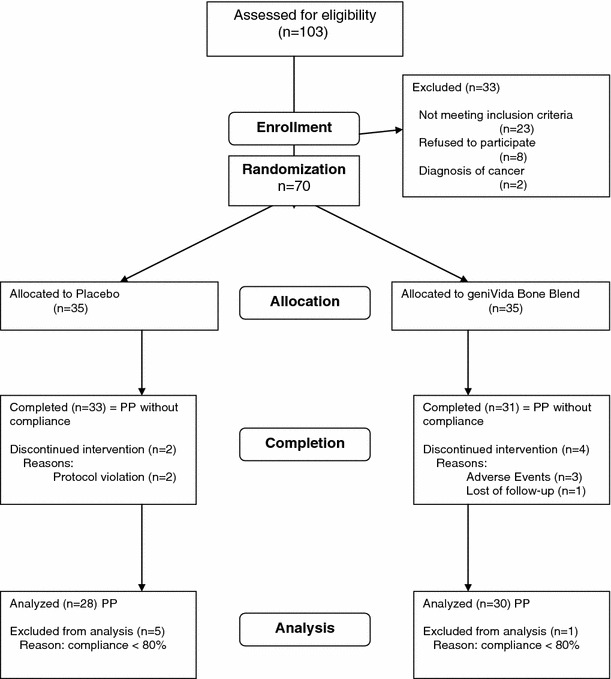
Subject disposition

Exclusion criteria were T-score <–2.5 at total hip and spine (either or both), body mass index (BMI) >30 or <21, use of HT within the previous 6 months, use of any drug that might interfere with bone metabolism within the previous 12 months, extreme dietary habits, use of dietary supplements while on study except multi-vitamins, total genistein blood concentrations >100 ng/mL measured at pre-study examination, unexplained weight loss or weight gain of >5 kg in the 3 months prior to the study, history of liver or pancreatic diseases, cardiovascular disease, history of breast cancer, endometrial cancer or other malignancy except basal and squamous cell skin cancer, history of thromboembolism, any fractures within the past year except for fingers, toes and facial bones, susceptibility to fractures, endometrial thickness >6 mm, endometrial polyps, insulin-dependent diabetes mellitus, any condition that might interfere with the absorption of the investigational product, co-medications. A total of 70 women were enrolled and randomly assigned to supplementation groups (Fig. 1).

### Measurements

#### Bone mineral density

BMD measurements at the femoral neck, lumbar spine, Ward’s triangle, trochanter and intertrochanter, total hip and whole body were made by dual-energy X-ray absorptiometry (DXA) with a Hologic 4500 instrument (Hologic Inc. Waltham, MA). The densitometers in the Osteoporosis Research Center were operated from a core densitometry laboratory by certified radiological technicians. Densitometry scans were performed according to Osteoporosis Research Center Standard Operating Procedures based on Hologic training. To obtain DXA measurements, subjects were placed in the supine position on a padded table while a scanning arm passed back and forth over their entire body. Radiation exposure was trivial and judged to be acceptable by the Internal Review Board.

The coefficient of variation of the Hologic 4500 at the Creighton University Osteoporosis Research Center is 1.1% at the spine and 1.3% at the hip. Measurements at baseline and 6 months were duplicated (with repositioning at each measurement), and the average of both measurements was used for statistical analyses.

#### Anthropometry, diet and physical activity

Body composition (fat mass and lean body mass) was calculated from DXA whole-body scans. Diets and physical activity were assessed via 3-day diaries. To complete the diet diary, subjects were asked to record everything they ate for 3 days (2 weekdays and 1 weekend day consecutive). The dietician reviewed with the subjects how to determine portion sizes. The 3-day diaries were analyzed by a dietician using The Food Processor Nutrition and Fitness Software (Version 7.8, 2001, ESHA Research, Salem, OR). Physical activity was assessed using a portion of the Paffenbarger activity questionnaire [25]. Subjects were asked to estimate the number of hours each day that they spent in various levels of physical activity.

#### Biological samples

*Bone markers*: Plasma bone-specific alkaline phosphatase (BAP) was determined using a Beckman Coulter Access Immunoassay System by Chemiluminescent Immunoassay, and urinary N-telopeptide (Ntx) was determined by Immunochemical method suing Ortho Diagnostics Vitros Analysis.

Total osteocalcin (OC) in plasma was determined by a solid-phase immuno-radiometric assay (Cis Bio International, France). Plasma concentration of undercarboxylated osteocalcin (ucOC) was determined by modification of the hydroxyapatite binding assay at Tufts University (Sarah Booth, Jean Mayer USDA Human Nutrition Research) [26]. Deoxypyridinoline (DPD) in urine was determined by enzyme immunoassay (EIA), and osteoprotegerin (OPG) and receptor activator of nuclear factor kappa B ligand (RANKL) were determined using an enzyme-linked immunoassay.

*Genistein*: Ten milliliter of venous blood was drawn into tubes containing EDTA and centrifuged at 3,000 rpm for 10 min at 4 °C. Plasma was pipetted and aliquoted into two propylene tubes (1.5 mL each) and stored at −20 °C. Free and total (sum of unconjugated and conjugated) genistein was determined by LC/MS at DSM Nutritional Products Ltd., Kaiseraugst, Switzerland.

*Phylloquinone*: Plasma vitamin K concentration was determined by reversed-phase high-performance liquid chromatography at Tufts University (Sarah Booth, Jean Mayer USDA Human Nutrition Research Center on Aging).

*25-hydroxyvitamin D3 (25(OH)D)*: Plasma 25(OH)D was measured by radioimmunoassay (RIA) at a certified clinical laboratory.

*EPA and DHA*: Serum EPA and DHA were measured by GC-FID and HPLC–MS (Agilent Q-TOF 6,530 High resolution mass spectrometer) at DSM Nutritional Products Ltd., Kaiseraugst, Switzerland.

*Clinical blood chemistry and urine analysis*: Plasma samples were used for the analysis of hormones (E2, parathyroid hormone, FSH, luteinizing hormone and thyroid stimulating hormone), lipids (total cholesterol, triglycerides, HDL, VLDL cholesterol, LDL cholesterol), hematology, HbA1c, coagulation factors, serology (hepatitis B and C) and thrombophilia screening. Urine electrolytes were determined by certified clinical laboratories according to clinically accepted standardized analytic protocols. Urine analysis (including microscopy) was performed in second spot urine.

### Adverse event (AE) monitoring

All AEs were reported in the case report form (CRF) and classified as mild, moderate or severe. In addition, all serious adverse events (SAEs) were reported to the Pharmacovigilance partner Contract Research Organization (CRO) UBC and the respective IRB. Subjects were instructed to contact the research nurses if they experienced an AE. Also, the nurses questioned the subjects at each visit about AEs. Medical records were obtained for any SAEs that involved medical follow-up. The research nurses followed each subject with an AE until the event was resolved. If treatment for AEs became necessary, the medication(s) were reported on the concomitant medication section of the CRF. AEs were presented in a frequency table by system organ class and intervention group. In addition, the nature, incidence, severity and cause for each AE were reported.

### Data quality assurances

Data quality was monitored by a professional monitor (RGB Consulting, Canada) and data management by the electronic data capture company (ClinIT, Germany).

### Power analysis and statistical analyses

#### Determination of sample size

The primary health outcomes of this study were the effects of GBB on change in BMD at the lumbar spine and femoral neck. Sample size power calculations were based on the premise that the standard deviation is not greater than double the effect (as percent of baseline) of intervention (see references for evidence in support of this premise) [27–34]. As a result of these calculations, the sample size was determined to be 30 completers per group. Assuming a dropout rate of ~15%, the final sample size was determined to be 35 per group; with this sample size, the trial had 75% power to detect a statistically significant effect with a type I error equal to 10% (trend).

#### Statistical analysis

Differences between the GBB and placebo groups were examined using both an ANOVA model (independent t test with equal variance) and a covariance model (ANCOVA, including baseline values measured at visit 1 as the covariate). Variables measured at all three visits were analyzed with the general linear model repeated measures ANOVA. Differences were considered significant if *p* < 0.05. Safety data were evaluated by descriptive statistics, and statistically significant differences were determined by *t* tests (within the groups by paired *t* test). As appropriate, further exploratory analyses were performed (Pearson’s correlations between concentrations of investigational products: genistein, phylloquinone, 25(OH)D and different efficacy and safety end-points), as well as stepwise regression analysis for determination of predictors of BMD change.

## Results

### Data set, subjects demographics and screening characteristics

Of the 70 subjects randomized, three withdrew before visit 2 and another three between visits 2 and 3. Therefore, 64 subjects were included in the per-protocol (PP) data set that does not take into consideration compliance. There were no statistically significant differences in baseline characteristics and demographics between groups with the exception that total cholesterol was significantly higher in the GBB group (Table 1). All subjects experienced natural menopause and had consumed no more than 2–3 dL of alcohol daily. About half of the subjects reported using multivitamins, but only one quarter reported taking them regularly. Six subjects were classified as non-compliant and were excluded from data analysis. Consequently, the PP analysis included 58 subjects (Fig. 1).

**Table 1:** Baseline characteristics and demographics at screening or baseline visits

Parameter	geniVida™ bone blend n = 31	Placebo n = 33	All + n = 64
Age (years)	54.8 ± 2.5	54.7 ± 2.3	54.7 ± 2.4
Body weight (kg)	68.0 ± 9.2	71.1 ± 9.0	69.6 ± 9.2
BMI (kg/m^2^)	24.9 ± 2.8	25.9 ± 2.8	25.4 ± 2.9
Fat mass (%)^a^	38.0 ± 6.0	36.8 ± 6.1	37.5 ± 6.1
Years since menopause (Y)	2.2 ± 0.8 (n = 30)	2.1 ± 0.8 (n = 32)	2.1 ± 0.8 (n = 62)
Hot flashes	24/31	27/33	51/64
Hot flashes since (Y)	5.4 ± 5.8 (n = 24)	8.1 ± 10.3 (n = 27)	6.8 ± 8.5 (n = 51)
T-score (lumbar spine)	−0.58 ± 1.06	−0.48 ± 1.33	−0.53 ± 1.20
T-score (hip)	−0.52 ± 0.86	−0.53 ± 0.84	−0.52 ± 0.84
Previous fractures more than 1 year before inclusion	0/31	0/33	0/64
Intake of concomitant medication	24/31	26/33	50/64
Intake of multivitamins^b^	15/31	14/33	29/64
Smokers	1/31 (max 10 cigarettes/day)	0/33	1/64
Systolic BP (mmHg)	114 ± 13	115 ± 11	114 ± 12
Diastolic BP (mmHg)	72 ± 9	73 ± 8	73 ± 9
Heart rate (bpm)	68 ± 7	67 ± 8	67 ± 7
Total cholesterol(mg/mL)	223.0 ± 35.4*	205.5 ± 29.0	214.0 ± 33.2
Estradiol (pg/mL)	10.2 ± 2.1	10.4 ± 3.3	10.3 ± 2.8
Follicle-stimulating hormone (mIU/mL)	109.9 ± 47.4	104.8 ± 33.7	107.3 ± 40.7

^a^at visit 1 (baseline); + data only include subjects who completed the study

^b^About 25% of the subjects reported taking them regularly (~200 IU vitamin D and 150–300 mg Ca)

* *p* < 0.5 compared to placebo

Reference ranges: Total cholesterol: <200 mg/mL

### Bone mineral density

**Fig. 2:**
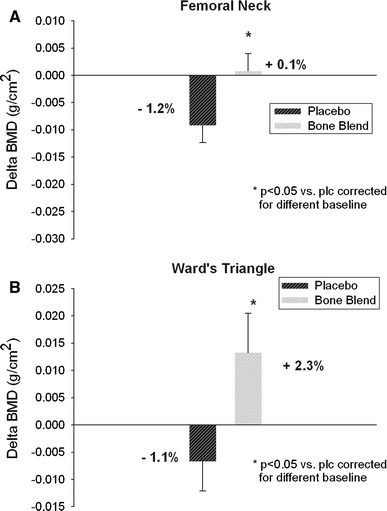
Change of bone mineral density (BMD; 6 months minus baseline) at femoral neck (**a**) and Ward’s triangle (**b**)

At the 6-month time point, subjects supplemented with the GBB (*n* = 30) maintained femoral neck BMD, whereas in the placebo group (*n* = 28), BMD significantly decreased (*p* = 0.007, Fig. 2a), resulting in a 1.3% difference between groups (*p* < 0.05). Similarly, as shown in Fig. 2b, there was a significant difference in BMD between the two groups at Ward’s triangle (+2.3 vs −1.1%, *p* < 0.05). The difference in femoral neck BMD between groups remained statistically significant in the intention to treat (ITT) analysis (*p* = 0.05); however, when baseline BMD was factored into the analysis, statistical significance was no longer quite achieved (*p* = 0.058). The BMD delta at Ward’s triangle, even when considering baseline BMD, maintained statistical significance in the ITT analysis (*p* = 0.002).

Lumbar spine BMD decreased slightly more in women given the placebo (−1.4%) than the GBB (−1.1%), but the difference between groups was not statistically significant (*p* = 0.55). Changes in BMD at the other sites measured (trochanter, intertrochanter, total hip and whole body) did not differ between groups (data not shown).

### Blood and urine analytes

**Fig. 3:**
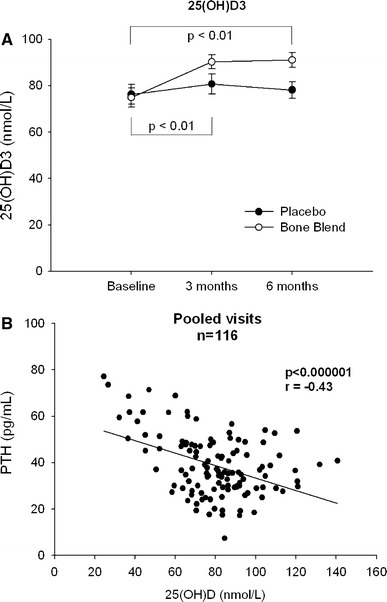
25(OH)VitD concentration course with statistical significance between GBB and placebo (**a**) and correlation to PTH (**b**)

At both the 3- and 6-month time points, both BAP and Ntx significantly increased in the GBB group in comparison with those in baseline and in the placebo group (Table 2). There were no other significant changes in bone markers (OC, ucOC, DPD, OPG and RANKL) nor did urinary calcium and phosphorus excretion differ between groups (data not shown). As expected, in comparison with baseline, total plasma genistein concentrations and EPA and DHA increased significantly in the GBB group at 3 and 6 months, whereas there was no change in the placebo group (Table 2). Although plasma phylloquinone concentrations increased from baseline to 3 months in the GBB group, at 6 months, values were lower than at baseline, but none of these changes were statistically significant. Although baseline plasma 25(OH)D concentrations were in the optimal range for both groups, suggesting that the subjects were generally fit [35], we observed an increase at 3 months in the GBB group that was maintained to the end of the study (Fig. 3a; Table 2). The plasma 25(OH)D remained stable in the placebo group. Parathyroid hormone (PTH) significantly decreased in the GBB group reflecting the increased plasma 25(OH)D concentration (Table 3).

**Table 2:** Blood analytes at different time points (PP considering compliance), mean (SD) and repeated measures ANOVA statistics

Parameter	geniVida bone blend (*n* = 30)			Placebo (*n* = 28)			*p* = GBB versus Plc	
	BSL	3 months (V2)	6 months (V3)	BSL	3 months (V2)	6 months (V3)	BSL/V2	BSL/V3
BAP (μg/L)	14.68 (4.64)	15.17 (4.38)	15.06 (4.87)	15.94 (3.96)	14.52 (3.03)	14.71 (3.93)	0.002	0.054
Ntx/crt (nM/mM crt)	40.73 (11.75)	41.33 (12.15)	45.23 (14.17)	44.46 (13.01)	44.04 (12.54)	41.46 (12.01)	ns	0.024
Genistein tot (ng/mL)	2.1 (9.7)	96.1 (88.1)	129.8 (179.7)	4.8 (14.5)	1.5 (4.4)	2.6 (5.6)	<0.001	<0.0001
25(OH)D (nM/L)	74.9 (23.0)	90.2 (16.5)	91.1 (17.8)	76.3 (22.2)	80.7 (23.1	78.1 (18.8)	0.009	0.009
Phylloquinone (nmol/L)	2.2 (3.1)	2.6 (2.9)	2.1 (2.1)	1.5 (2.2)	1.6 (1.3)	1.2 (0.9)	0.07	ns
EPA (μg/100μL)	2.87 (1.06)	Not determined	7.16 (2.86)**	4.17 (4.28)	Not determined	4.09 (4.05)	na	0.003*
DHA (μg/100μL)	7.29 (2.71)	Not determined	11.17 (4.16)**	9.35 (5.20)	Not determined	8.34 (5.59)	na	<0.05*

*BSL* Baseline, V2: 3-month visit, V3: 6-month visit, Reference range: BAP: 0–22.4 μg/L, *ns* nonsignificant, *na* not applicable, *25(OH)D* 25-hydroxyvitamin D3, *EPA* Eicosapentaenoic acid (C20:5n-3), *DHA* Docosahexaenoic acid (C22:6n-3)

* Significant at V3 versus placebo

** Significant versus BSL (*p* < 0.0001)

**Table 3:** Blood analytes at different time points (PP, safety population), mean (SD)

Parameter	geniVida bone blend (*n* = 31)		*p* = V3 versus scr^a^	Placebo (*n* = 33)		*p* = V3 versus scr^a^	scr *p* = GBB versus Plc^b^	V3 *p* = GBB versus Plc^b^
	Screening	6 months (V3)		Screening	6 months (V3)			
TC (mg/mL)	223.0 (35.4)	207.8 (28.5)	*p* = 0.014	205.5 (29.0)	195.9 (25.7)	*p* = 0.002	*p* = 0.033	ns
LDL-C (mg/mL)	130.3 (33.0)	122.4 (31.7)	ns	118.0 (26.7)	115.1 (24.2)	ns	ns	ns
HDL (mg/mL)	72.3 (15.9)	67.7 (16.1)	*p* = 0.014	68.8 (16.3)	62.1 (13.4)	<0.0001	ns	ns
Estradiol								
(pg/mL)	10.2 (2.1)	11.5 (3.9)	*p* = 0.044	10.4 (3.3)	10.7 (2.3)	ns	ns	ns
PTH (pg/mL)	42.4 (13.8)	38.0 (11.4)	*p* = 0.015	37.8 (13.3)	37.8 (14.5)	ns	ns	ns

*Scr* screening, V3: 6-month visit, Reference ranges: TC: <200 mg/mL; LDL-C: <100 mg/mL; HDL: 40–59 mg/mL; Estradiol: <20 pg/mL; PTH: 12–88 pg/mL, *ns* nonsignificant, *TC* total cholesterol, *PTH* parathyroid hormone

^a^Paired *t* test

^b^Unpaired *t* test

The season of the year during which subjects were enrolled, which could reflect differences in sun exposure and therefore endogenous vitamin D synthesis, had no effect on the change in BMD at any bone site. Stepwise regression analysis revealed that baseline BMD was the strongest predictor of BMD after 6-month supplementation; baseline 25(OH)D did not further predict BMD. The second most predictive factor determining BMD was GBB independent of the season when subjects were enrolled. This observation suggests that supplementation was sufficient to counterbalance seasonal variations in vitamin D concentrations. In addition, there was a significant relationship between plasma 25(OH)D and PTH at screening/baseline and visit 3 (pooled data: Fig. 3b). There were no statistically significant correlations at any of the three time points between any blood analytes and BMD or bone markers.

### Anthropometry and dietary intake

There were no significant changes in body weight, BMI and body fat mass in either group when comparing baseline with final values. Similarly, there were no changes in macro- or micronutrient intakes (Table 1). Average daily intake of vitamin D3, calcium and n-3 fatty acids ranged from 100 to 140 IU, 700 to 800 mg and 700 mg—4 g (1–6% from total fat intake), respectively. According to the dietary assessment, isoflavone intake was zero.

### Safety

*Laboratory findings*: Clinical chemistry, hematology, lipids, coagulation factors, hormones and urine analysis (including microscopy) were analyzed in all subjects who completed the full dosage regimen (*n* = 64). Total cholesterol and LDL-C were above the normal reference range at screening in both groups (Table 3), and HDL levels were higher than the medium reference range. Although some single determinations slightly deviated from normal values, most laboratory parameters were well within the normal reference range. Of note, E2 increased in the GBB group by 12.7% (*p* = 0.044), but remained within the normal range (Table 3).

*Endometrial Thickness*: At no time point did endometrial thickness (ET) differ between the groups. However, ET significantly decreased in the GBB group between screening and study end (2.3–1.8 mm, *p* = 0.007), whereas there was no change in the placebo group (2.2–2.3 mm, *p* = 0.62) and no statistical significant difference observed between GBB and placebo at study end.

*ECG and vital signs*: There were no statistically significant changes in vital signs or ECG recordings during the course of the study in any subject, and in no case did any change raise clinical concern.

### Tolerability

*Summary of adverse events:* There were no significant differences in AEs between groups. Of the total of 59 AEs that occurred in 35 subjects, 37 were reported in 20 subjects in the placebo group and 22 in 15 subjects in the GBB group. One AE in each of three individuals led to their withdrawal from the study, and 51% of AEs led to treatment. None of the AEs were classified as severe, 66.1% were considered mild and 33.9% as moderate. All of the AEs except one were considered to be unrelated to the trial supplement (98%). One moderately severe AE (abdominal pain) was judged as probably related to the GBB supplement. After withdrawal from the trial during the second month, this symptom resolved. Approximately 80% of the AEs resolved at study completion.

AEs were distributed over a wide range of system organ classes (SOC); however, most of the AEs fell into the SOC of “Infections and infestations” and “Reproductive system and breast disorders.” The most frequently reported AE was vaginal hemorrhage (vaginal bleeding and spotting); 5 AEs of this type occurred in 4 subjects in the placebo group compared to one each in 2 subjects in the GBB group.

*Serious Adverse Event (SAE)*: There were three cases of incidental findings on the final vaginal ultrasounds, two in one subject in the placebo group and one in the intervention group. These SAEs were documented as Unanticipated Events and unrelated to the effects of the GBB by the investigator who reported to the IRB.

## Discussion

The results of this pilot study show that GBB can reduce bone loss in early postmenopausal women. In the placebo group, women lost 1.2 and 1.1% BMD at the femoral neck and Ward’s triangle, respectively, whereas women in the GBB group gained 0.1 and 2.3% at these sites, respectively. The bone maker results were unexpectedly inconsistent. We observed increases in both BAP and Ntx at both the 3- and 6-month time points in the GBB group, whereas no changes were noted in the placebo group. This was remarkable since another bone formation marker (OC) and one bone resorption marker (DPD) remained unchanged. In general, the bone turnover marker BAP is decreased during bone-sparing osteoporosis therapies, for example, bisphosphonates [36], hormone therapies [37] or selective estrogen receptor modulars (SERMs). Interestingly, only the more sophisticated therapy, using intermittent application of a recombinant human PTH (1–34) that can reverse bone loss, is also accompanied by increased BAP levels [38]. We speculate that the increased bone turnover is linked to genistein initiating bone formation [39], which was evident after just 3 months. Bone formation is coupled to a parallel bone resorption response, which is observed by the increased Ntx level after 6 months. Therefore, it is unclear why the bone formation marker OC and the resorption marker DPD apparently remained unchanged in the treatment group. An increase in OC and a reduction in DPD were observed in the genistein supplementation study of Morabito et al. [29] using a daily 54 mg dose. Interestingly, when using 200 μg/days vitamin K1 alone for 6 weeks, Bügel et al. [40] did not observe any effect on bone turnover markers (total osteocalcin, BAP, Ntx and DPD). Nevertheless, the vitamin K status markers were improved after supplementation. The serum ucOC/cOC ratio was significantly decreased. Interestingly, Schurgers et al. [41] could improve the serum carboxylated/undercarboxylated osteocalcin ratio with even a lower dose of 100 μg vitamin K1. Our study included healthy subjects that were taking a balanced diet on the top of the GBB supplementation. Obviously, the 150 μg vitamin K1 had no measurable impact on their K status. As expected, plasma levels of genistein, EPA and DHA and 25(OH)D increased in the active group. That there were significant effects at the femoral neck and Ward’s Triangle is especially notable because the sample size for this study was calculated to detect a trend, not necessarily statistically significant effects. Therefore, the results indicate that a follow-up study requires a sample size of only 70 subjects to confirm the effects of the GBB at these two bone sites. However, the results generated from this pilot study also indicate that to detect a statistically significant effect (*p* < 0.05) at the lumbar spine would require a sample size of 900 subjects.

The findings of the present pilot study are consistent with the majority of the published positive isoflavone supplementation trials that also struggled with the limitations of small sample size (<50 subjects/group) and short duration (≤1 years) [13–15]. Intriguingly, the four large isoflavone studies that lasted longer than 2 years resulted in ambiguous outcomes [16–19]. In the study by Marini et al. [16], there were dramatic increases in postmenopausal spinal and hip BMD in response to 54 mg/days genistein in aglycone format. On the other hand, Levis et al. and Alekel et al. [17, 18] that used mixtures of isoflavones in glycoside format observed only modest or no effect at all. Both groups used high doses of isoflavones, 80 and 120, and 200 mg/days, respectively. The contrasting results trigger speculations that the different application formats, aglycone vs glycoside, could be responsible for the results due to different bioavailability [42–44]. However, most recent research now casts doubts on this hypothesis. Tai et al. [19] administered 300 mg isoflavones in aglycone format and yet did not observe skeletal benefits. An alternative explanation is that genistein by itself functions differently than genistein in combination with daidzein and glycitein. Currently, there is little evidence to support this theory, however. Another possibility could be that 80 mg and higher doses of genistein are not beneficial; consequently, the bone-sparing effect of genistein is negated. The in vitro data of Dang et al. [45] point in this direction. They have shown that isoflavones stimulate osteogenesis at low concentrations and inhibit osteogenesis at high concentrations in osteoblasts and osteoprogenitor cells [45], which could explain the observed discrepancy.

Although the current results are consistent with some previous trials showing genistein exerts skeletal benefits, there are important differences. For example, during the first year of the 3-year trial by Marini et al. [16], women in the genistein group gained 2.4% BMD at the femoral neck, whereas women in the placebo group lost approximately this much bone. There were also marked increases in spinal BMD in response to genistein. The more pronounced effect observed in that trial could be because the subjects were osteopenic and as such had much lower baseline BMD (e.g., femoral neck BMD, 0.0667 vs 0.740) than women in the current trial). However, if the women in the placebo group in the current trial continued to lose BMD at their 6-month rate over the course of 1 year, their bone loss would have essentially matched the loss in the Italian study. Therefore, differences in baseline BMD may not have contributed to differences between the two studies. An alternative and more straightforward explanation is that the lower dose used in the current study (30 vs 54 mg/days) accounts for the less robust results. Animal data suggest this could be the case [46]. Differences in dose may also explain why the current study did not show effects at the spine whereas the study by Marini et al. [16] did. We believe that a longer duration trial is required to see significant effect of the GBB on spinal BMD.

It is notable that GBB supplementation had a greater effect on Ward’s triangle than it did on the femoral neck. Bone loss at Ward’s triangle in the placebo group was similar to that of the femoral neck, whereas there was a 2.3% increase in the GBB group. Ward’s triangle was not measured in the study by Marini et al. [16]. However, in an earlier study from this group, the effect of genistein on Ward’s triangle was slightly greater than it was on the femoral neck. Ward’s triangle is not a true anatomic area but is generated by the DXA scan as the area having the lowest BMD in the femoral head. Interestingly, a small study by Yoshihashi et al. [47] found that in men, the only DXA BMD measurement that was sensitive for detecting osteoporosis was Ward’s triangle. Among the women in the current study, the DXA BMD at Ward’s triangle and the femoral neck were equally sensitive in detecting changes in BMD.

Because a combination of ingredients was used, it is not possible to determine to what extent the individual components contained in the GBB contributed to the observed skeletal benefits. Combined calcium and vitamin D supplementation has been shown to reduce fracture risk in postmenopausal women to a greater extent than supplementation with either agent alone [48]. In the current study, the baseline plasma 25(OH)D concentrations were in the normal range. As expected, only GBB supplementation further increased the 25(OH)D level. Each group was supplemented with calcium (500 mg), which when added to their dietary intake brought their total calcium intake to >1,200 mg/days. Interestingly, despite the rather small study sample size, it was still possible to detect an inverse relationship between plasma vitamin D and PTH levels (Fig. 3b), which is consistent with previously published data [21, 49].

In 2006, Cockayne et al. [50] reviewed the fracture risk reduction after vitamin K supplementation in a meta-analysis. They showed that phylloquinone and menaquinone-4 reduced bone loss and fracture risk. A more recent review by Iwamoto et al. [22] also concluded that vitamin K supplementation reduces fracture risk. However, most trials included in this analysis used far higher doses than the amount contained within the GBB [51]. Furthermore, according to these authors, vitamin K works via mechanisms other than by increasing BMD and affecting bone turnover. Thus, even if the inclusion of vitamin K enhanced the ability of the GBB to reduce fracture, the benefits may have not been detected in the health outcomes analyzed. The fact that there was no change in the undercarboxylated/carboxylated osteocalcin ratio also argues against vitamin K contributing to the increases in BMD that were observed.

Finally, in regard to omega-3 fatty acids, the evidence that they exert skeletal benefits is intriguing but still quite speculative [51–53]. Nevertheless, in ovariectomized rats, Krammer [54] found that genistein (15 mg/kg bw) and n-3 PUFA (5% by weight) independently increased femoral BMD over an 8-week period and the combined effect was greater than the effect of either agent alone. Our study has taken advantage of this finding and used nutritional levels of PUFA and genistein in combination with vitamin D and K in the GBB supplementation mixture. The results suggest that genistein supplementation at levels that are compatible with Asian isoflavone intake exerts favorable effects on bone health although long-term trials are needed to confirm efficacy.

The GBB was well tolerated by the subjects as there were few AE and no SAE attributable to the intervention. Plasma levels of E2 did increase although modestly so and they remained within the normal range, which is generally consistent with the literature showing that isoflavones have little effect on estrogen levels [55]. Furthermore, and more importantly, there were no effects of the GBB on endometrial thickness. In fact, endometrial thickness decreased over time in the GBB group although the difference in final values between groups was not significant. Estrogen markedly stimulates endometrial tissue and increases risk of endometrial cancer [56]. In the previously mentioned 3-y trial by Marini et al. [16], genistein (54 mg/d) had no effect on endometrial thickness in postmenopausal women.

In conclusion, the results of this pilot study suggest that the use of the physiological relevant dose of genistein in combination with EPA and DHA and vitamins D3 and K1 (GBB) may help to prevent osteoporosis and may reduce fracture risk, at least at the hip, in postmenopausal women. The results are also reassuring about the safety of this product. However, additional research and especially longer-term clinical trials are needed before definitive conclusions can be made.
